# Balancing anatomical limits and dynamic adaptation: Understanding the determinants of successful vaginal breech delivery

**DOI:** 10.1016/j.eurox.2026.100453

**Published:** 2026-03-15

**Authors:** Maria Serena Rothkamm, Sabine Katharina Maschke, Lena Steinkasserer, Diane Renz, Anna Lena Biermann, Lena Radomsky, Vivien Dütemeyer, Constantin von Kaisenberg, Peter Hillemanns, Lars Brodowski

**Affiliations:** a Department of Obstetrics, Gynecology and Reproductive Medicine, Hannover Medical School, Hannover, Germany; b Department of Diagnostic and Interventional Radiology, Hannover Medical School, Hannover, Germany

**Keywords:** Breech presentation, Mode of delivery, MR Pelvimetry, Mechanics of birth

## Abstract

**Objective:**

To evaluate the association between obstetric parameters, MRI pelvimetry findings, and delivery outcomes in term singleton breech pregnancies attempting vaginal birth.

**Methods:**

This retrospective observational study included 80 women with singleton breech presentations who underwent MRI pelvimetry and attempted vaginal delivery at Hannover Medical School between August 2021 and December 2024. The primary outcome was the mode of delivery (vaginal vs. intrapartum cesarean). Secondary analyses explored associations between obstetric parameters, neonatal outcomes, and the need for obstetric maneuvers. Statistical analyses included univariable logistic regression and receiver operating characteristic (ROC) curve assessment, to provide descriptive context for the observed univariable associations.

**Results:**

Of 80 cases, 57 (71.3%) achieved vaginal delivery and 23 (28.7%) required intrapartum cesarean section. Women with vaginal delivery had significantly larger pelvimetric dimensions, including the obstetric conjugate (12.9 vs. 12.4 cm, p = 0.009) and pubic angle (95.8° vs. 91.6°, p = 0.020). In descriptive ROC analyses, intrapartum course variables showed higher discriminatory values within the study sample. Prolonged oxytocin augmentation (unadjusted p = 0.009; AUC = 0.80; BH-adjusted p = 0.204) and extended active first stage of labor (unadjusted p < 0.001; AUC = 0.77; BH-adjusted p = 0.026) were associated with cesarean delivery; however, after adjustment for multiple testing, only the duration of the active first stage remained statistically significant. No association was found between birth weight and head circumference —whether estimated by ultrasound or measured after birth—, or fetal leg position and successful vaginal delivery. In the vaginal delivery group, adverse neonatal outcomes correlated with longer rupture of membranes-to-birth intervals and prolonged first and second stages of labor (p < 0.05), whereas pelvimetric measures showed no significant association.

**Conclusion:**

In term breech deliveries, intrapartum dynamics showed stronger associations with intrapartum cesarean and neonatal outcomes than MRI pelvimetric dimensions, whereas fetal anthropometric parameters and leg position did not show comparable associations. MRI pelvimetry offers anatomical information at baseline, while continuous intrapartum evaluation reflects dynamic processes that are more closely associated with observed outcomes in vaginal breech birth.

## Introduction

Breech presentation occurs in approximately 3–4% of all singleton term pregnancies and continues to pose a challenge in modern obstetrics [1]. Despite ongoing advances in perinatal care, the optimal mode of delivery for fetuses in breech position remains a matter of discussion. Earlier studies and guideline recommendations have contributed to a general preference for cesarean section in many settings. However, accumulating evidence suggests that vaginal breech birth can represent a safe alternative under well-defined conditions and in the presence of experienced obstetric teams [2], [3], [4], [5].

Successful vaginal breech delivery depends on multiple interrelated factors influencing the mechanics of labor. These may include maternal pelvic dimensions, fetal biometry and position, uterine contractility, and the skillful application of obstetric maneuvers [6], [7]. Comprehensive pre-delivery assessment and careful intrapartum monitoring are therefore essential to minimize maternal and neonatal risks [8]. In this context, imaging-based magnetic resonance (MRI) pelvimetry has been explored as an adjunct tool to evaluate pelvic adequacy and support individualized birth planning. MRI provides non-invasive and reproducible measurements of relevant pelvic parameters such as the obstetric conjugate, intertuberous distance, and pubic angle, which may contribute to estimating the likelihood of vaginal delivery success [9], [10], [11]. Nevertheless, the predictive value of MRI pelvimetry remains under discussion, and its role should be considered alongside additional obstetric and perinatal factors.

The increasing global rate of cesarean deliveries underscores the importance of refining selection criteria for women eligible for a trial of labor in breech presentation. Identifying those who are most likely to achieve favorable outcomes through vaginal delivery could reduce unnecessary surgical interventions and associated maternal morbidity while maintaining neonatal safety.

The present retrospective study aimed to analyze the association between obstetric parameters, MRI pelvimetry findings, and delivery outcomes in term singleton pregnancies with breech presentation attempting vaginal birth. Furthermore, secondary analyses sought to explore factors related to labor progress, the need for obstetric maneuvers, and neonatal outcomes. By integrating clinical, anamnestic, and imaging data, this study seeks to improve understanding of the determinants influencing the success of vaginal breech delivery and to provide evidence to support individualized counseling and delivery planning.

## Methods

### Study design and data collection

A retrospective observational study was conducted at Hannover Medical School, Germany. Between August 2021 and December 2024, anamnestic, clinical, and perinatal data were collected from singleton pregnancies with breech presentation that resulted in vaginal delivery attempt at the institution and after undergoing MRI pelvimetry. Data were extracted from the hospital’s electronic patient management system after discharge.

The primary outcome was the mode of delivery (vaginal delivery vs. intrapartum cesarean section). The secondary outcome was to explore associations between obstetric parameters and selected outcomes in the group of successful vaginal delivery, including the composite neonatal outcome the time interval from breech to cephalic delivery, and the frequency of obstetrical maneuvers. Composite neonatal outcome was defined as appearance of at least one of the following components: Admission to the neonatal intensive care unit (NICU), umbilical artery pH < 7.1, 5-minute Apgar score < 7, need for neonatal resuscitation, mask ventilation or oxygen supplementation. Umbilical artery pH < 7.1 was used as a marker of intrapartum hypoxia [12]. A 5-minute Apgar score < 7 and the need for neonatal resuscitation indicated impaired neonatal transition [13], [14]. Mask ventilation or oxygen supplementation reflected respiratory compromise at birth [15]. NICU admission served as a global indicator of neonatal morbidity [16]),

A total of 80 singleton term pregnancies in breech presentation were included in the analysis, comprising 57 vaginal deliveries and 23 intrapartum cesarean sections.

Healthy women with uncomplicated singleton breech pregnancies who were able to choose their mode of delivery after informed counseling and attempt for vaginal delivery were included. Exclusion criteria comprised planned cesarean section based on patients’ choice or medical indication (e.g., placenta praevia, pre-eclampsia with severe features, history of more than one cesarean section, abnormal fetal Doppler findings or severe fetal growth restriction, severe maternal illness, or major fetal malformation).

All patients received standard obstetric care. After clinical examination and sonographic fetometry, available options for mode of delivery were discussed. Risks and potential complications of both vaginal and cesarean delivery were explained, along with the feasibility and success rate of external cephalic version based on current literature and institutional experience.

MRI pelvimetry was offered to all women who did not immediately opt for cesarean section following counseling. The examination was performed from 36 + 0 weeks of gestation. MRI was explained as a non-invasive cross-sectional imaging technique without ionizing radiation and therefore safe for both mother and fetus [17]. It was particularly recommended for primiparous women but not mandatory for attempting vaginal birth.

**Fig. 1:**
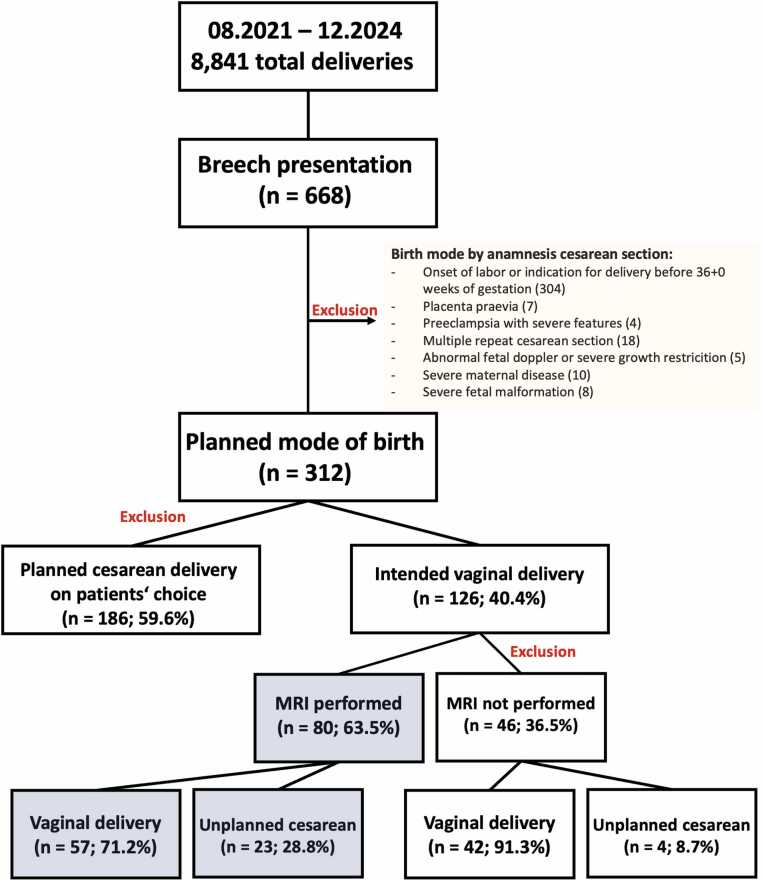
Flowchart of singleton breech pregnancies during the study period (August 2021–December 2024), showing exclusions, distribution of delivery mode, and delivery outcomes according to MRI pelvimetry among women intending vaginal delivery. Percentages refer to proportions within each subgroup.

To assess the feasibility of vaginal delivery, the obstetric conjugate, intertubal distance, and pubic angle were compared with published reference values and institutional experience [9], [18]. Patients were informed that MRI pelvimetry provides supportive data but must be interpreted in conjunction with other factors such as fetal biometry, fetomaternal Doppler results, and maternal history. Based on the results and counseling, women proceeded with either a planned trial of labor or an elective cesarean section. During the study period, 8841 deliveries were recorded, including 668 breech presentations (7.6%). Of these, 312 had no contraindications for vaginal delivery. A trial of vaginal breech delivery was attempted in 126 cases (40.4%), of which 80 (63.5%) underwent MRI pelvimetry, resulting in 99 successful vaginal births (78.6%). Fig. 1 illustrates a flowchart showing the case selection from all deliveries during the study period to the final analytic cohort of term singleton breech pregnancies, including eligibility criteria and reasons for exclusion.

### Magnetic resonance imaging (MRI)

All MRI examinations were performed on a 1.5 T scanner (Avanto, Siemens Healthineers, Erlangen, Germany) in accordance with the American College of Radiology guidelines for safe and optimal fetal MRI [17]. MRI examinations included axial and coronal T1-weighted turbo spin echo, axial true fast imaging with steady-state free precession (FISP), and sagittal T2-weighted half-acquisition single-shot turbo spin echo sequences of the maternal pelvis and abdomen (slice thickness 3–5 mm), with a total acquisition time of approximately 15 min. All examinations were supervised by a radiologist and a radiographer before, during, and after scanning.

### Statistical analysis

Statistical analyses were performed using GraphPad Prism nine software (GraphPad Software Inc.) and IBM SPSS Statistics 28 (IBM SPSS Software).

Continuous variables are presented as medians with interquartile range (IQR), and categorical variables as absolute frequencies and percentages. Data distribution was assessed using the Shapiro–Wilk normality test. Group differences between delivery modes were analyzed using the Mann–Whitney U-test or unpaired *t*-test for continuous variables, as appropriate, and the Chi-square test for categorical variables. Variables showing statistical significance were further examined using univariable logistic regression to assess their crude associations with the primary outcome. To account for multiple model testing, p-values were adjusted using the false discovery rate (FDR) according to Benjamini–Hochberg (BH adj.) for single primary outcome (intrapartum cesarean delivery). Analyses of neonatal outcomes were exploratory and outcome specific. Therefore, no multiplicity adjustment was applied, and results are interpreted as hypothesis-generating. Odds ratios (ORs) were derived from logistic regression models. Sensitivity, specificity, ROC curves, and AUC values were used descriptively to illustrate the ability of the examined variables to discriminate outcomes within the study sample. Univariable logistic regression analyses were additionally performed to explore associations between obstetric parameters and selected outcomes, including the composite neonatal outcome, the time interval from breech to cephalic delivery, and the frequency of obstetrical maneuvers. Continuous variables were entered individually into separate models. Considering the limited sample size and number of events, we refrained from multivariable regression modeling to avoid overfitting and unstable effect estimates. Given the collinearity of several obstetric and MRI parameters, univariable logistic regression analyses were deemed the most appropriate and statistically approach for the present cohort. All reported associations are based on univariable (crude) logistic regression analyses; no adjusted estimates are presented. Only variables with p < 0.05 were visualized.

## Ethics

This study was approved by the local Ethics Committee of the Hannover Medical School and conducted according to the principles of the Declaration of Helsinki (approval number: 10629_BO_K_2022).

## Results

A total of 80 singleton term pregnancies in breech presentation were included, comprising 57 vaginal deliveries and 23 intrapartum cesarean sections. Maternal age, height, body mass index, and parity were comparable between both groups. The median gestational age at delivery was higher in women who underwent cesarean section (40.0 weeks [39.3–40.4]) compared to those with vaginal delivery (39.4 weeks [38.9–39.7]; p = 0.041). Preexisting and gestational diabetes were rare and showed no group differences. Neonatal birthweight, head circumference, and APGAR scores were comparable. The arterial cord pH was higher in the cesarean group (7.27 vs. 7.21; p = 0.037) (Table 1).

**Table 1:** Maternal characteristics and Neonatal characteristics and outcome.

Characteristics	Vaginal Delivery from Breech N = 57	Intrapartum Cesarean N = 23	P-Value
*Maternal characteristics*			
Maternal age (years)	31 [29–34]	34 [30–37]	0.198
Maternal height (cm)	168 [165 – 174]	166 [163 – 171]	0.083
Nulliparous (%)	44 (77.2)	19 (82.6)	0.592
Gestational age at delivery (weeks of gestation)	39.4 [38.9 – 39.7]	40.0 [39.3 – 40–4]	**0.041**
Pre-pregnancy Body Mass Index (kg/m^2^)	23.0 [19.7 – 25.7]	24.1 [20.7 – 25.3]	0.673
Preexisting Diabetes Mellitus (%)	1 (1.8)	0 (0)	0.523
Development of Gestational Diabetes (%)	4 (7.0)	1 (4.3)	0.655
Previous cesarean birth (%)	0 (0)	1 (4.3)	0.113

*Neonatal characteristics and outcome*			
Head circumference (cm)	35 [34.5 – 36.3]	35.5 [35 – 36.2]	0.515
Head circumference percentile	56 [41–81]	63 [32–86]	0.858
Birthweight (kg)	3.35 [3.05 – 3.63]	3.26 [3.13 – 3.77]	0.438
Birthweight percentile	44 [19–63]	36 [17–70]	0.966
APGAR (at 5th min)	10 [9,10]	10 [9,10]	0.555
APGAR (at 5th min) < 7	3 (5.3)	0 (0)	0.262
APGAR (at 5th min) < 4	0 (0)	0 (0)	N.a.
Arterial pH – value	7.21 [7.15 – 7.26]	7.27 [7.21 – 7.31]	**0.037**
Arterial pH – value < 7.1	3 (5.3)	2 (8.7)	0.566
Resuscitation (%)	2 (3.5)	0 (0)	0.363
Oxygen administration (%)	10 (17.5)	7 (30.4)	0.202
Non-invasive respiratory support (%)	9 (15.8)	5 (21.7)	0.526
Transfer to the neonatal unit (%)	9 (15.8)	1 (4.3)	0.161
Neonatal unit > 4 days (%)	5 (8.8)	1 (4.3)	0.497

Description of study collective (n = 80) divided by mode of delivery. Continuous variables are expressed as medians [interquartile range] and categorical variables as absolute frequencies (percentage). Distribution was examined with Shapiro-Wilk normality test. For normally distributed variables, comparisons were performed using the unpaired *t*-test, for non-normally distributed variables, the Mann–Whitney U-test was applied. The chi-square test was used to compare categorical variables. A P-value < 0.05 was considered as significant and are shown in bold.

Magnetic resonance pelvimetry demonstrated several significant differences in pelvic dimensions. The obstetric conjugate was larger in the vaginal delivery group (12.9 cm [12.5–13.5]) compared with the cesarean group (12.4 cm [12.0–12.9]; p = 0.009). Pelvic width (13.7 vs. 13.3 cm; p = 0.034), sacral pelvic outlet (11.6 vs. 11.2 cm; p = 0.031), coccygeal outlet (9.1 vs. 8.5 cm; p = 0.041), and pubic angle (95.8° vs. 91.6°; p = 0.020) were also significantly larger among women with vaginal delivery. Other pelvic parameters and all fetal sonographic measurements, including biparietal diameter, head circumference, and estimated fetal weight, were not significantly different between groups (Table 2).

**Table 2:** MR pelvimetry and most recent sonographic estimation before birth.

Characteristics	Vaginal Delivery from Breech N = 57	Intrapartum Cesarean N = 23	P-Value
*MR pelvimetry*			
Obstetrical conjugate (cm)	12.9 [12.5 – 13.5]	12.4 [12.0 – 12.9]	**0.009**
Pelvic width (cm)	13.7 [13.3 – 14.4]	13.3 [12.8 – 13.9]	**0.034**
Sacral pelvic outlet diameter (cm)	11.6 [11.2 – 12.3]	11.2 [10.7 – 11.7]	**0.031**
Coccygeal pelvic outlet (cm)	9.1 [8.4 – 9.8]	8.5 [8.0 – 9.5]	**0.041**
Pelvic aperture angle (°)	91.8 [83.3 – 102.2]	89.4 [85.1 – 96.0]	0.514
Pelvic inlet angle (°)	143.2 [130.6 – 151.8]	143.0 [136.2 – 149.6]	0.853
Pelvic inclination (°)	63.3 [59.5 – 67.9]	64.7 [60.1 – 69.2]	0.409
Interspinious distance (cm)	11.3 [10.6 – 11.8]	11.1 [10.3 – 11.6]	0.257
Intertuberous distance (cm)	13.0 [12.2 – 13.6]	12.5 [11.8 – 13.1]	0.068
Pubic angle (°)	95.8 [91.5 – 99.9]	91.6 [88.5 – 97.2]	**0.020**
Biparietal diameter (cm)	9.6 [9.3 – 9.9]	9.4 [9.2 – 9.7]	0.295
Occipitofrontal diameter (cm)	12.3[11.8 – 12.6]	12.1 [11.7 – 12.6]	0.828
Head circumference (cm)	34.6 [33.7 – 35.8]	34.5 [33.1 – 35.8]	0.493
Gestational age at pelvimetry (weeks)	37.3 [36.6 – 37.9]	37.4 [37.0 – 37.7]	0.680
Time interval between pelvimetry and birth (days)	15 [9.5 – 19]	19 [11–24]	0.156

*Most recent sonographic estimation before birth*			
Biparietal diameter (cm)	9.3 [9.1 – 9.7]	9.4 [9.0 – 9.7]	0.845
Occipitofrontal diameter (cm)	12.2 [11.9 – 12.5]	12.2 [12.0 – 12.5]	0.697
Head circumference (cm)	33.9 [33.2 – 34.8]	34.4 [33.3 – 34.8]	0.461
Head circumference percentile	71 [54–85]	75 [51–87]	0.703
Abdominal circumference (cm)	33.9 [32.2 – 34.9]	33.4 [33.3 – 35.0]	0.595
Abdominal circumference percentile	50 [33–62]	44 [37–64]	0.904
Estimated fetal weight (kg)	3.29 [3.00 – 3.65]	3.20 [3.14 – 3.65]	0.761
Estimated fetal weight percentile	47 [31–72]	43 [29–71]	0.786
Gestational age at ultrasound (weeks)	38.9 [38.1–39.2]	39.0 [38.4 – 39.3]	0.320
Time interval between last ultrasound and birth (days)	5 [2], [3], [4], [5], [6], [7], [8], [9]	4 [3], [4], [5], [6], [7], [8], [9], [10], [11]	0.453

Description of study collective (n = 80) divided by mode of delivery. Continuous variables are expressed as medians [interquartile range] and categorical variables as absolute frequencies (percentage). Distribution was examined with Shapiro-Wilk normality test. For normally distributed variables, comparisons were performed using the unpaired *t*-test, for non-normally distributed variables, the Mann–Whitney U-test was applied. The chi-square test was used to compare categorical variables. A P-value < 0.05 was considered as significant and are shown in bold.

Prelabor rupture of membranes occurred in 36.8% of vaginal deliveries and 69.6% of cesarean deliveries (p = 0.008). The interval between rupture of membranes and birth was longer in the cesarean group (730 min [396–981]) than in the vaginal group (219 min [67–743]; p = 0.008). The duration of the active first stage of labor was prolonged in the cesarean group (255 min [125–413] vs. 80 min [46–218]; p < 0.001). The time of oxytocin augmentation was also longer (173 min [109–265] vs. 55 min [30–127]; p = 0.009) (Table 3).

**Table 3:** Characteristics of birth and obstetrical management.

Characteristics	Vaginal Delivery from Breech N = 57	Intrapartum Cesarean N = 23	P-Value
Fetal leg position Frank breech (%) Complete or incomplete breech (%)	44 (77.2) 13 (22.8)	19 (82.6) 4 (17.4)	0.592
Bishop score at arrival in labor ward	10 [9,10]	10 [9,10]	0.882
Prelabor rupture of membranes (%)	21 (36.8)	16 (69.6)	**0.008**
Cervical dilatation at timepoint of rupture of membranes (cm)	4 [2–9]	2 [2–4]	0.075
Time interval between rupture of membranes and birth (min)	219 [67 – 743]	730 [396 – 981]	**0.008**
Induction of labor (%)	38 (66.6)	14 (60.9)	0.623
Time interval of start of induction of labor to active first stage of labor (min)	1480 [255 – 2353]	1673 [1006 – 3164]	0.236
Duration of active first stage of labor / cervical dilatation of 5 cm to total cercival dilatation (min)	80 [46 – 218]	255 [125 – 413]	**< 0.001**
Second stage of labor (total in min)	56 [29 – 110]	70 [43 – 145]	0.250
Duration of active second stage of labor / active pushing (min)	11 [7–25]	10 [7–23]	0.789
Oxytocin augmentation during labor (%)	32 (56.1)	17 (73.9)	0.140
Time of oxytocin augmentation during labor (min)	55 [30 – 127]	173 [109 – 265]	**0.009**
Epidural analgesia (%)	13 (22.8)	10 (43.5)	0.064
Cervical dilatation at timepoint of implementation of epidural analgesia (cm)	6 [4–8]	4 (4 – 9]	0.964
Cesarean section Secondary Emergency*		20 (87.0) 3 (13.0)	
Time interval of breech to cephalic birth (min)	2 [2–4]		
Rate of total obstetrical maneuvers (%)	41 (71.9)		
Lovset maneuver (%)	6 (10.5)		
Bickenbach`s arm delivery (%)	4 (7.0)		
Mueller maneuver (%)	7 (12.3)		
Frank nudge maneuver (%)	1 (1.8)		
Bracht maneuver (%)	18 (31.6)		
Veit-Smellie maneuver (%)	9 (15.8)		
Birth in an upright position (%)	9 (15.8)		
Perineal injury Episiotomy 1^st´^ perineal tear 2nd´ perineal tear 3rd´and 4th´ perineal tear	21(36.8) 11 (19.3) 6 (10.5) 1 (1.8)		

Description of study collective (n = 80) divided by mode of delivery. Continuous variables are expressed as medians [interquartile range] and categorical variables as absolute frequencies (percentage). Distribution was examined with Shapiro-Wilk normality test. For normally distributed variables, comparisons were performed using the unpaired *t*-test, for non-normally distributed variables, the Mann–Whitney U-test was applied. The chi-square test was used to compare categorical variables. A P-value < 0.05 was considered as significant and are shown in bold. *defined as immediate indication for delivery due to high risk for fetal asphyxia with general anesthesia.

In the logistic regression analysis statistical different values underwent univariable analysis to identify variables with an association with intrapartum cesarean. Prolonged oxytocin augmentation (p = 0.009), longer active first stage of labor (p < 0.001), smaller pelvic measurements including obstetric conjugate (p = 0.013), pelvic width (p = 0.041), and pubic angle (p = 0.025), higher gestational age (p = 0.046), and prelabor rupture of membranes (p = 0.015) were significantly associated with intrapartum cesarean delivery.

**Fig. 2:**
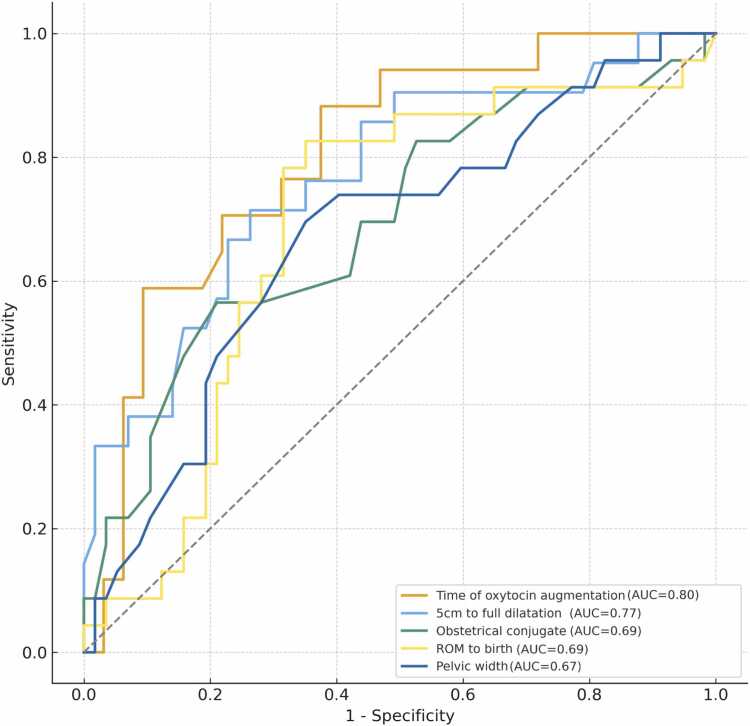
Receiver operating characteristic (ROC) curves of the five variables with the numerically highest area under the curve (AUC) values in relation to intrapartum cesarean delivery. All variables demonstrated poor to modest discriminatory ability (AUC < 0.70) and did not remain statistically significant after false discovery rate (FDR) adjustment. The ROC analyses describe discrimination within the observed dataset and are not intended to support clinical prediction. Only variables with p < 0.05 in the univariable analyses are shown for illustrative purposes; all tested variables are reported in the corresponding tables.

Receiver operating characteristic (ROC) curves were generated for descriptive purposes to illustrate the discriminatory characteristics of selected intrapartum variables within the study cohort (Fig. 2). The duration of oxytocin administration (AUC = 0.80) and the duration of the active first stage of labor (AUC = 0.77) showed the numerically highest AUC values. However, as these variables represent intrapartum course characteristics that are closely linked to the clinical decision-making process, the ROC analysis is intended solely to describe discrimination within the observed dataset and does not imply prospective predictive utility. Given the number of parallel model tests performed, control of the false discovery rate (FDR) was applied using the Benjamini–Hochberg (BH) procedure. After BH adjustment, only the duration of the active first stage of labor remained statistically significant (BH-adjusted p = 0.026), whereas oxytocin augmentation (BH-adjusted p = 0.204), obstetric conjugate (BH-adjusted p = 0.204), pelvic width (BH-adjusted p = 0.288), gestational age (BH-adjusted p = 0.288), prelabor rupture of membranes (BH-adjusted p = 0.204), coccygeal pelvic outlet (BH-adjusted p = 0.288), pubic angle (BH-adjusted p = 0.265), rupture-to-birth interval (BH-adjusted p = 0.334), and sacral pelvic outlet diameter (BH-adjusted p = 0.334) did not retain statistical significance (Table 4).

**Table 4:** Logistic regression analysis examining associations with intrapartum cesarean delivery.

Variable	OR	95% CI	p- Value	AUC	BH adj.
Time of oxytocin augmentation during labor (hour)	1.1	1.00–1.22	0.009	0.80	0.204
Duration of active first stage of labor / cervical dilatation of 5 cm to total cercival dilatation (hour)	1.1	1.00–1.21	< 0.001	0.77	0.026
Obstetrical conjugate (cm)	0.4	0.20–0.82	0.013	0.69	0.204
Time interval between rupture of membranes and birth (hour)	1.2	1.00–1.30	0.078	0.69	0.334
Pelvic width (cm)	0.6	0.33–0.97	0.041	0.67	0.288
Gestational age at delivery (weeks of gestation)	1.9	1.01–3.67	0.046	0.66	0.288
Prelabor rupture of membranes (yes)	3.6	1.29–10.25	0.015	0.65	0.204
Sacral pelvic outlet diameter (cm)	0.7	0.41–1.06	0.088	0.65	0.334
Coccygeal pelvic outlet (cm)	0.6	0.35–0.98	0.045	0.65	0.288
Pubic angle (°)	0.9	0.84–0.98	0.025	0.64	0.265

Variables showing statistically significant differences were subjected to univariable analysis to assess their crude associations with the primary outcome. Due to multiple model tests, adjusted p-values according to the false discovery rate by Benjamini–Hochberg (BH adj.) were additionally indicated. Odds ratios (ORs) with a 95% confidence interval (95% CI) were obtained from the logistic regression analysis. Odds ratios for continuous variables are calculated per one-unit increase as indicated in parentheses. The diagnostic accuracy of each model was assessed through sensitivity, specificity, receiveroperating characteristics (ROC) curves, and the area under the ROC curve (AUC).

**Fig. 3:**
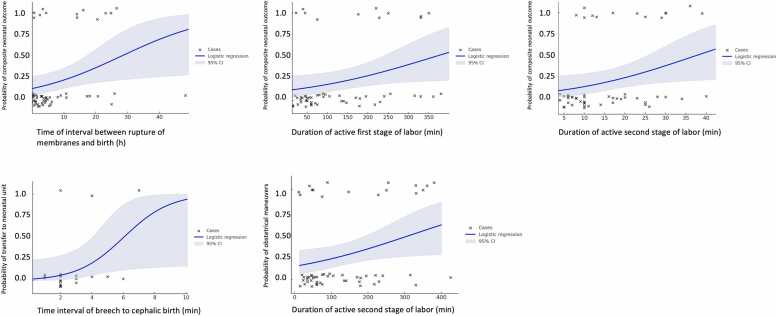
Fitted logistic regression curves with 95% confidence intervals.

Univariable logistic regression analyses were performed to assess associations between observed obstetric parameters and selected outcomes in the vaginal birth group. Longer rupture-to-birth intervals (p = 0.019), prolonged active first stage of labor (p = 0.042), extended active second stage of labor (p = 0.042), and longer breech-to-head intervals (p = 0.041) were associated with adverse neonatal outcomes, whereas none of the pelvimetric measurements reached significant differences (Table 5; Fig. 3).

**Table 5:** Logistic regression model examining associations with an adverse outcome during vaginal birth (n = 57).

Variable	Depentent variable	OR	95% CI	p- Value
Composite neonatal outcome	Time interval between rupture of membranes and birth (hour)	1.07	1.01 – 1.13	0.019
	Duration of active first stage of labor / cervical dilatation of 5 cm to total cercival dilatation (hour)	1.01	1.0 – 1.01	0.042
	Duration of active second stage of labor / active pushing (min)	1.06	1.0 – 1.13	0.042

Time interval of breech to cephalic birth (min)	Transfer to the neonatal unit (yes/no)	1.96	1.01 – 3.81	0.041

Rate of total obstetrical maneuvers (n)	Duration of active first stage of labor / cervical dilatation of 5 cm to total cercival dilatation (hour)	1.01	1.0 – 1.1	0.043

Univariable logistic regression analyses were performed to assess associations between observed obstetric parameters and selected outcomes (composite neonatal outcome, time interval of breech to cephalic birth and rate of total obstetrical maneuvers). Continuous variables were entered individually into separate models. Odds ratios (OR), 95% confidence intervals (95% CI), and p-values were calculated using maximum likelihood estimation. Odds ratios for continuous variables are calculated per one-unit increase as indicated in parentheses. Only variables with p < 0.05 were visualized. Absolute event numbers for each component of composite neonatal outcome can be find in supplementary Table 1.

## Discussion

In this retrospective single-center cohort of term singleton breech pregnancies attempting vaginal birth after MRI pelvimetry, we observed that intrapartum dynamics, particularly duration of oxytocin augmentation and length of the active first stage, were most strongly associated with intrapartum cesarean delivery and showed higher AUC values within the study sample (AUC 0.80 and 0.77, respectively). Given that these variables are closely linked to the clinical definition and management of dystocia and arise during labor, their discriminatory capacity likely reflects their proximity to the decision-making process rather than independent predictive ability. Several pelvic measurements, namely obstetric conjugate, pelvic width, coccygeal outlet and pubic angle were larger among women who achieved vaginal birth, however, their discriminatory performance with respect to intrapartum cesarean was modest. Importantly, ROC analyses were used for descriptive and exploratory purposes only and were not intended to support clinical prediction, decision-making, or the derivation of clinical thresholds. While these several pelvimetric parameters differed statistically between women who achieved vaginal birth and those who required intrapartum cesarean delivery, their overall discriminatory performance was limited. The observed AUC values for MRI-derived measurements remained below thresholds generally considered clinically useful, and these variables did not retain statistical significance after adjustment for multiple testing. Consequently, MRI pelvimetry cannot reliably discriminate between vaginal delivery and intrapartum cesarean section at the individual patient level. Rather than serving as a standalone tool for outcome prediction, pelvimetry should be interpreted as providing baseline anatomical context that may complement, but not replace, dynamic intrapartum assessment and clinical judgment. These findings underscore the importance of integrating structural, clinical, and temporal factors when evaluating the likelihood of successful vaginal breech birth.

In the subset of successful vaginal births, adverse neonatal outcomes were associated with longer rupture of membranes to birth interval, prolonged active first stage of labor, extended active second stage of labor, and longer breech-to-head interval, whereas pelvimetric parameters did not show significant associations with neonatal outcomes.

These findings highlight that labor mechanics in breech presentation are determined by a complex interplay of maternal, fetal, and intrapartum factors. While MRI pelvimetry can help identify women with less favorable pelvic dimensions, labor progression and management-related variables were more closely associated with delivery outcomes within this cohort, although these parameters become available only after labor onset. This observation is in line with previous research showing that adequate labor progress is crucial for successful vaginal breech delivery [7], [19], [20], [21]. The higher rate of prelabor rupture of membranes among women who required intrapartum cesarean and the association between prolonged rupture of membranes -to-birth intervals and neonatal morbidity suggest that membrane status and timing are clinically relevant factors in breech labor [22], [23]. However, it should be noted that a large retrospective cohort study revealed no differences in maternal and fetal outcomes with regard to the timing of membrane rupture in vaginally planned or completed breech deliveries at term [24]. Delayed progression after rupture of membranes may predispose to fetal compromise and necessitate operative intervention. Likewise, the observed relationship between prolonged first and second stages of labor and both intrapartum cesarean delivery and adverse neonatal outcomes is consistent with prior studies showing that extended active phases increase the risk of fetal hypoxia and the need for obstetric maneuvers [20], [21].

In contrast, fetal biometry and head circumference were not associated with mode of delivery or neonatal morbidity, which aligns with prior studies indicating that, once appropriate case selection and intrapartum management by experienced teams are ensured, fetal size alone does not predict poor outcomes [6], [25]. These findings highlight the importance of a dynamic intrapartum assessment in vaginal breech birth. MRI pelvimetry provides valuable structural information, but real-time labor parameters and fetal well-being ultimately determine the success of vaginal delivery. A combined approach integrating pelvimetric, clinical, and temporal parameters seem most appropriate to guide individualized decision-making. MRI pelvimetry may therefore serve as a useful adjunct during counseling, particularly in identifying women with abnormal pelvic parameters, yet its results should always be interpreted within a broader clinical context that includes parity, fetal position, and institutional expertise. Studies demonstrated that MRI offers non-invasive and reproducible measurements of pertinent pelvic parameters, which may facilitate the estimation of the probability of vaginal delivery success [6], [9]. Furthermore, MRI pelvimetry has the capacity to influence the decision-making process of pregnant women regarding the desired mode of delivery [26].

The strengths of this study include a well-defined and homogeneous patient cohort, standardized MRI acquisition with detailed multi-parameter pelvimetric measurements, and comprehensive documentation of intrapartum time intervals, enabling direct comparison of static pelvic anatomy with dynamic labor characteristics. The use of false discovery rate adjustment further strengthens the robustness of statistical inference. Nevertheless, interpretation of our findings must be considered in the context of several limitations. This was a retrospective, single-center study conducted in a tertiary care setting with specific expertise in vaginal breech delivery, which may limit the generalizability of the results. MRI pelvimetry was not mandatory and was performed in a selected subgroup, potentially introducing selection bias. In addition, the sample size—particularly for analyses of neonatal outcomes—was limited, restricting statistical power and the feasibility of fully adjusted multivariable models. Consequently, most associations are based on unadjusted analyses and should be interpreted as exploratory rather than causal. Although false discovery rate correction was applied to account for multiple testing, the risk of residual type I error cannot be completely excluded. The neonatal outcome analysis was based on a small number of events (n = 13), and the resulting odds ratios should therefore be interpreted as exploratory and potentially unstable. MRI pelvimetry was performed only in a selected subgroup, and the final analytic cohort represents a highly filtered population managed at a specialized, high-volume center with institutional expertise in vaginal breech delivery. This selection process likely inflates observed vaginal delivery success rates and limits the external validity of our findings. Consequently, these results should be interpreted with caution and should not be directly extrapolated to low-volume or non-specialist centers, where patient selection, clinical experience, and intrapartum management strategies may differ considerably.

Although several pelvimetric dimensions differed statistically between women who achieved vaginal birth and those who underwent intrapartum cesarean delivery, effect sizes were small, overlap between groups was substantial, and discriminatory performance was modest. Accordingly, MRI pelvimetry cannot reliably discriminate between vaginal and intrapartum cesarean delivery at the individual patient level. While intrapartum parameters showed stronger associations with delivery outcomes in this cohort, these variables become available only after labor onset and therefore cannot inform initial decision-making regarding mode of birth. Accordingly, the ROC analyses should not be interpreted as evidence of early clinical prediction, but rather as descriptive measures of discrimination within the observed dataset. MRI pelvimetry may nonetheless contribute to baseline anatomical assessment and risk stratification when interpreted within a broader clinical context, rather than as a standalone tool for outcome discrimination. Future research should focus on prospective, multicenter validation of predictive models that combine anatomic and intrapartum variables. Incorporating real-time labor data such as cervical dilation rate, uterine activity, and fetal descent could improve the accuracy of individualized risk assessment. Standardized management of prelabor rupture of membranes and well-defined time thresholds for intervention could also contribute to optimizing outcomes. Furthermore, differentiating individual neonatal outcomes rather than composite indices may allow a more nuanced understanding of perinatal risks associated with breech vaginal birth.

## Conclusion

In term breech pregnancies attempting vaginal birth, intrapartum parameters were more closely associated with intrapartum cesarean delivery and showed numerically higher discrimination within the study sample than individual pelvimetric measures. Prolonged labor duration and extended oxytocin augmentation should be interpreted as markers of evolving dystocia or fetal compromise rather than as causal determinants of intrapartum cesarean delivery. Although certain pelvic dimensions were more favorable among women who achieved vaginal birth, these anatomical measures were not associated with adverse neonatal outcomes in this cohort and estimated fetal weight likewise did not show an independent association with intrapartum cesarean delivery or neonatal morbidity. In contrast, longer intrapartum intervals were more closely associated with neonatal morbidity. Taken together, these findings support a complementary interpretation in which MRI pelvimetry provides baseline anatomical context, while intrapartum assessment and labor dynamics appear more closely related to observed maternal and neonatal outcomes.

## CRediT authorship contribution statement

**Maria Serena Rothkamm:** Writing – original draft, Visualization, Methodology, Formal analysis, Data curation. **Lena Steinkasserer:** Writing – review & editing, Investigation, Data curation. **Sabine Katharina Maschke:** Writing – review & editing, Validation, Formal analysis, Data curation. **Anna Lena Biermann:** Writing – review & editing, Investigation. **Diane Renz:** Writing – review & editing, Formal analysis. **Vivien Dütemeyer:** Writing – review & editing, Investigation, Data curation. **Lena Radomsky:** Writing – review & editing, Investigation. **Peter Hillemanns:** Writing – review & editing, Supervision. **Constantin von Kaisenberg:** Writing – review & editing, Supervision. **Lars Brodowski:** Writing – review & editing, Writing – original draft, Supervision, Methodology, Conceptualization.

## Declaration of Competing Interest

The authors declare the following financial interests/personal relationships which may be considered as potential competing interests: Lars Brodowski reports article publishing charges was provided by Hannover Medical School. If there are other authors, they declare that they have no known competing financial interests or personal relationships that could have appeared to influence the work reported in this paper

## Footnotes

Appendix A Supplementary data associated with this article can be found in the online version at doi:10.1016/j.eurox.2026.100453.

## References

[1] Hofmeyr G.J., Kulier R., West H.M. (2015). External cephalic version for breech presentation at term. Cochrane Database Syst Rev.

[2] Hannah M.E., Hannah W.J., Hewson S.A., Hodnett E.D., Saigal S., Willan A.R. (2000). Planned caesarean section versus planned vaginal birth for breech presentation at term: a randomised multicentre trial. Term Breech Trial Collab Group Lancet.

[3] Hofmeyr J., Hannah M. (2006). Five years to the Term Breech Trial: the rise and fall of a randomized controlled trial. Am J Obstet Gynecol.

[4] ACOG Committee Opinion No. 745: Mode of Term Singleton Breech Delivery. Obstetrics and gynecology. 2018;132(2):e60-e3. . 10.1097/AOG.0000000000002755 30045211

[5] AWMF S3-Leitlinie Die Sectio caesarea. Deutsche Gesellschaft für Gynäkologie und Geburtshilfe ev (DGGG). 2020. .

[6] Zander N., Raimann F.J., Al Naimi A., Bruggmann D., Louwen F., Jennewein L. (2022). Combined assessment of the obstetrical conjugate and fetal birth weight predicts birth mode outcome in vaginally intended breech deliveries of primiparous women-a frabat study. J Clin Med.

[7] Azria E., Le Meaux J.P., Khoshnood B., Alexander S., Subtil D., Goffinet F. (2012). Factors associated with adverse perinatal outcomes for term breech fetuses with planned vaginal delivery. Am J Obstet Gynecol.

[8] Goffinet F., Carayol M., Foidart J.M., Alexander S., Uzan S., Subtil D. (2006). Is planned vaginal delivery for breech presentation at term still an option? Results of an observational prospective survey in France and Belgium. Am J Obstet Gynecol.

[9] Klemt A.S., Schulze S., Bruggmann D., Louwen F. (2019). MRI-based pelvimetric measurements as predictors for a successful vaginal breech delivery in the Frankfurt Breech at term cohort (FRABAT). Eur J Obstet Gynecol Reprod Biol.

[10] Franz M., von Bismarck A., Delius M., Ertl-Wagner B., Deppe C., Mahner S. (2017). MR pelvimetry: prognosis for successful vaginal delivery in patients with suspected fetopelvic disproportion or breech presentation at term. Arch Gynecol Obstet.

[11] van Loon A.J., Mantingh A., Serlier E.K., Kroon G., Mooyaart E.L., Huisjes H.J. (1997). Randomised controlled trial of magnetic-resonance pelvimetry in breech presentation at term. Lancet.

[12] Low J.A. (1997). Intrapartum fetal asphyxia: definition, diagnosis, and classification. Am J Obstet Gynecol.

[13] Casey B.M., McIntire D.D., Leveno K.J. (2001). The continuing value of the Apgar score for the assessment of newborn infants. N Engl J Med.

[14] Wyckoff M.H., Aziz K., Escobedo M.B., Kapadia V.S., Kattwinkel J., Perlman J.M. (2015). Part 13: neonatal resuscitation: 2015 American heart association guidelines update for cardiopulmonary resuscitation and emergency cardiovascular care. Circulation.

[15] Wyckoff M.H., Wyllie J., Aziz K., de Almeida M.F., Fabres J., Fawke J. (2020). Neonatal life support: 2020 international consensus on cardiopulmonary resuscitation and emergency cardiovascular care science with treatment recommendations. Circulation.

[16] Crilly C.J., Haneuse S., Litt J.S. (2021). Predicting the outcomes of preterm neonates beyond the neonatal intensive care unit: what are we missing?. Pedia Res.

[17] website ACoR. ACR-SPR practise parameter for the safe and optimal performance of fetal magnetic resonance imaging (MRI). American College of Radiology. 2015. .

[18] Maschke S.K., Steinkasserer L., Renz D., von Kaisenberg C., Hillemanns P., Brodowski L. (2025). Maternal and neonatal short-term outcome after vaginal breech delivery >36 weeks of gestation with and without MRI-based pelvimetric measurements: a Hannover retrospective cohort study. J Perinat Med.

[19] Su M., McLeod L., Ross S., Willan A., Hannah W.J., Hutton E. (2003). Factors associated with adverse perinatal outcome in the Term Breech Trial. Am J Obstet Gynecol.

[20] Macharey G., Gissler M., Ulander V.M., Rahkonen L., Vaisanen-Tommiska M., Nuutila M. (2017). Risk factors associated with adverse perinatal outcome in planned vaginal breech labors at term: a retrospective population-based case-control study. BMC Pregnancy Childbirth.

[21] Macharey G., Ulander V.M., Heinonen S., Kostev K., Nuutila M., Vaisanen-Tommiska M. (2017). Risk factors and outcomes in "well-selected" vaginal breech deliveries: a retrospective observational study. J Perinat Med.

[22] Cattin J., Roesch M., Bourtembourg A., Maillet R., Ramanah R., Riethmuller D. (2016). [Obstetrical prognosis of breech presentations with premature rupture of membranes at term]. J Gynecol Obstet Biol Reprod (Paris).

[23] Sunarno I., Riu D.S., Mappaware N.A. (2021). Factors associated with and mode of delivery in prelabour rupture of membrane at secondary health care. Gac Sanit.

[24] Rasch E., Hentrich A.E., Kampf A.K., Deuster E., Hoock S.C., Louwen F. (2025). Influence of premature rupture of membranes on the peripartum outcomes in vaginally intended breech deliveries at term: a FRABAT study. Int J Gynaecol Obstet.

[25] Jennewein L., Theissen S., Pfeifenberger H.R., Zander N., Fischer K., Eichbaum C. (2021). Differences in biometric fetal weight estimation accuracy and doppler examination results in uncomplicated term singleton pregnancies between vertex and breech presentation. J Clin Med.

[26] Ebeling A.E., Maschke S.K., Holthausen-Markou S., Steinkasserer L., Klapdor R., Renz D. (2024). The influence of MRI-based pelvimetric measurements in mother's choice of delivery in fetal breech position. Arch Gynecol Obstet.

