# Supplementary material for: Balancing anatomical limits and dynamic adaptation: Understanding the determinants of successful vaginal breech delivery
Source: Eur J Obstet Gynecol Reprod Biol X. 2026 Mar 15;30:100453. doi: 10.1016/j.eurox.2026.100453 (PMC13014956; doi:10.1016/j.eurox.2026.100453)
Supplement: Supplementary file 1 — Supplementary material [file mmc1.docx]

**Supplementary Table 1**. Components of the composite neonatal outcome

**Neonatal outcome component n (%)**

Any composite neonatal outcome 13 (22.8)

NICU admission 9 (15.8)

Umbilical artery pH < 7.1 3 (5.3)

5-minute Apgar score < 7 3 (5.3)

Neonatal resuscitation 2 (3.5)

Mask ventilation 9 (15.8)

Oxygen supplementation 10 (17.5)*

**All neonates receiving oxygen supplementation also required mask ventilation, except for one case.*
